# More Knowledge and Thought Wanted

**Published:** 1887-07-09

**Authors:** 


### MORE KNOWLEDGE AND THOUGHT WANTED!

Having been engaged in hospital work nearly all my life, I should be glad to bear testimony to the great need of supporting all institutions for the sick and needy. It is sad and painful to think of beds being empty for want of funds. It is not for want of means funds are not forthcoming, but want of thought. When we hear of 25,000 people giving a guinea each on one day to see an American show, can we believe they are unable to wash out the stain now resting on our charities? I am sure if the non-residents in hospitals knew the work that is accomplished, the ceaseless toil of the surgeons and staff, no one would hesitate to give to the utmost extent of his or her means. No class work so hard as surgeons in hospitals,—never sure of any certain rest, always bright, cheerful, and ready night or day to the call of duty, that they should be surrounded with every comfort is most essential in their trying position, and that they should be able to command all they require, without the constant reminder that the funds will not admit of this or that, is but just. Soldiers and many others are inundated with honours, soldiers generally for the number of lives lost ; for saving life and alleviating pain you do not see the gold cross or diamond star. Personally, I believe they do not care for it, they work for such high and pure motives. May the coming appeal be abundantly responded to, and that we may hear no more of beds being empty for want of funds, is my earnest and sincere prayer.

Selhurst Road, South Norwood. L. B.

